# Assessing the impact of land use land cover change on regulatory ecosystem services of subtropical scrub forest, Soan Valley Pakistan

**DOI:** 10.1038/s41598-022-14333-4

**Published:** 2022-06-16

**Authors:** Gul Zareen Ghafoor, Faiza Sharif, Memuna Ghafoor Shahid, Laila Shahzad, Rizwan Rasheed, Amin Ul Haq Khan

**Affiliations:** 1 Sustainable Development Study Centre, Government College University Lahore, Lahore, Pakistan; 2 Department of Botany, Government College University Lahore, Lahore, Pakistan

**Keywords:** Ecology, Ecosystem ecology, Ecosystem services, Forest ecology, Forestry

## Abstract

This study investigated the effect of land use land cover (LULC) changes on carbon sequestration in the Hayat-ul-Mir subtropical scrub reserve forest, Pakistan. Supervised maximum likelihood classification of Landsat satellite imagery was done to assess spatio-temporal changes in LULC during 2007, 2013 and 2019. The CA–Markov model was used to simulate LULC of 2030. Spatial LULC data and carbon pools data was processed in Integrated Valuation of Ecosystem Services and Tradeoffs (InVEST) carbon model to investigate the effect of LULC on future carbon dynamics. The analysis revealed increase in cover of *A. modesta* and *O. ferruginea* and decrease in agriculture, built up and barren area of forest during 2007–2019 and 2030. The analysis also showed that the forest would additionally sequester 111 Mg C with an overall Net Present Value of $4112.05 in year 2030. The analysis revealed LULC changes on 25% area with increase and decrease in the value of ecosystem service (at some location) from carbon storage and loss as CO_2_ emissions respectively depending on the type of LULC converted. The study is helpful in identifying areas of potential carbon sequestration to maximize net benefits from management interventions.

## Introduction

Forests provide diverse regulatory, provisioning, supporting and cultural ecosystem services^1^. These services maintain the sustainability of human and ecological communities by providing food, timber, shelter, clean water and air, nutrient cycling, recreation, flood protection and regulate climatic processes^2^. Terrestrial ecosystems are documented to store annually net 2.0 Pg to 3.4 Pg of carbon contributing their considerable role in maintaining the regional and global carbon fluxes^3^. However, these ecosystems are directly affected by land use land cover (LULC) changes induced by socioeconomic drivers^4^. Rising global greenhouse gas concentrations also play their pivotal role in changing land cover by favoring the growth of one species and restricting the growth of others^5,6^. The considerable loss of tropical forests released 12.5% of the total anthropogenic CO_2_ emissions and reduced the economic value of carbon sequestration by 29% during 1997 to 2011^7^.

LULC changes are related to land management practices which may deplete or increase ecosystem carbon storage depending on the type and extent of the land cover converted^8^. For instance, conversion of forest to agriculture land increases food supply, but reduces carbon sequestration and increases the chances of soil erosion^9^. Therefore assessment of changes in forest cover and its carbon sequestration potential are crucial for appropriate land use planning, and to gain maximum benefits from conservation efforts. In this regard, integrated modeling approaches are now widely used to simulate and predict the impact of LULC changes on forest carbon stock^8^.

Integrated Valuation of Ecosystem Services and Tradeoffs (InVEST) model is one of the most widely used approaches to determine the spatial distribution and magnitude of multiple ecosystem services^8^. InVEST carbon model helps in mapping and quantifying current ecosystem carbon stock, but limits its application to compute carbon sequestration rates (SR) and its monetary benefits for the future due to lack of an in-built simulation module. Therefore, LULC simulation models such as Cellular Automata-Markov (CA-Markov) or others can be integrated with InVEST to provide predicted LULC spatial data to compute the future carbon SR and its benefits under LULC change scenario^10,11^.

A number of studies have been conducted on quantification and mapping of ecosystem carbon storage and sequestration under LULC change scenarios. Leh et al.^12^ reported decrease in carbon storage due to changes in the cover of agroforests (− 5%), cropland (− 52%) and forests (− 6%) and bare area in West Africa during 2000–2009. Liang et al.^10^ assessed spatio-temporal impacts of LULC change on carbon storage in Northwest China and reported 0.8% increase in oasis carbon storage during 2000–2009 but decrease in it by 0.93% during the simulated period (2009 to 2018). Sil et al.^8^ reported increase in carbon storage and sequestration during observed (1990–2006) and simulated (2006–2020) land cover in a mountain river basin of Portugal. Zhao et al.^13^ reported an increase in carbon storage by 13.8% due to LULC changes in Qinghai-Tibet Plateau during 2001–2010. Simulation of LULC changes in the mountain-oasis desert ecosystem in China (2015–2035) showed an overall increase in carbon sequestration service, but some decrease in it at certain locations in the mountain and oasis zone^14^. Zhao et al.^11^ predicted increase in carbon storage by 10.27 Tg under LULC changes from the ecological engineering program during 2015–2029 in Northwest China. Li et al.^15^ predicted 1.36% increase in the forest area and carbon storage of Beijing China during 2015 to 2030 under LULC changes. Hoque et al.^6^ reported 0.07% increase in the climate regulation service in Bangladesh during 1999–2019. Liu et al.^16^ documented increase in ecosystem carbon storage from 2.38 (10^8^ tons/year) to 2.45 (10^8^ tons/year) during 1985 to 2020 in Beijing, China due to increase in the area of woodland.

In Pakistan very few studies have been conducted on the assessment of ecosystem carbon stock while research on the impacts of LULC changes on ecosystem carbon storage is lacking. Nizami^17^ estimated 31.18 Mg/ha and 24.36 Mg/ha carbon stored in the Kherimurat and Sohawa subtropical scrub forests. Ghafoor et al.^18^ estimated 8.53 Mg/ha and 10.92 Mg/ha carbon stored in the bi-climax community (*Acacia modesta* and *Olea ferruginea* respectively) while Siddiq et al.^19^ reported overall 49.82 Mg/ha carbon stored in the Hayat-ul-Mir subtropical scrub forest in Pakistan. Ali et al.^20^ reported 8.34 MtC stored in the subtropical broadleaved forest in Khyber Pakhtunkhawa Province in Pakistan. Mannan et al.^21^ reported 313.94 Mg/ha, 221.34 Mg/ha and 131.7 Mg/ha carbon stored in the Himalayan moist temperate, subtropical chirpine and broadleaved forests in Pakistan during 1998–2018 and estimated the loss of 31.33 Gg/ha/year of stored carbon due to LULC changes during simulated (2028) period.

Present study was conducted on Hayat-ul-Mir (HM) subtropical scrub reserve forest in the Soan valley of district Khushab Pakistan. The subtropical scrub forest in Pakistan extends over an area of 1.3 Mha (0.3 Mha in Punjab alone) in the foothills and the lower slopes of the Himalayas, Kala Chitta, Suleman and Salt ranges^18^. Over a time span of 100 years, about 75% cover of the scrub forests in Pakistan has been lost and degraded due to socioeconomic activities and climatic changes^22^. But very little work has been done on assessment of carbon stock of subtropical forests in Pakistan and on impact of LULC changes on ecosystem services. To the best of our knowledge, Mannan et al.^21^ is the only study in Pakistan reporting impact of LULC changes on Himalayan forest carbon stock, but none has reported it for the subtropical scrub forests.

Hence, the current study was designed to assess the (1) spatial distribution of carbon storage in the HM subtropical scrub forest and, (2) impact of potential LULC changes on tree carbon storage and sequestration service in the near future. This is a reserve forest and is under state control so it was hypothesized that carbon sequestration will increase in future considering current management practices.

## Materials and methods

### Study area

**Figure 1:**
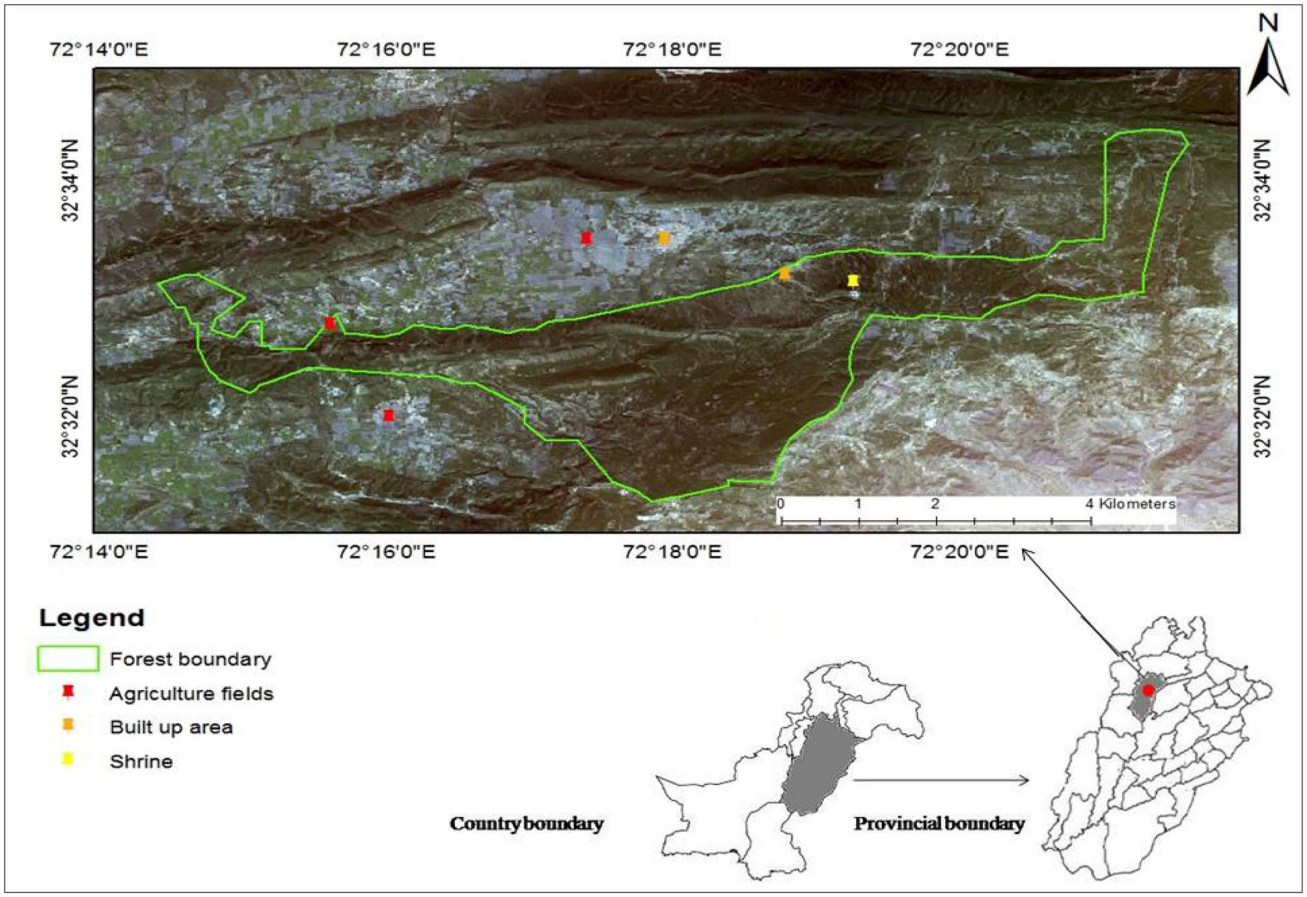
The location of the study area (*Source*: Satellite Imagery downloaded from USGS—Earth Explorer website, processed using ArcMap v. 10.2).

Hayat-ul-Mir (HM) subtropical scrub reserve forest in Soan Valley Pakistan located at 32.54° N and 72.31° E is stretched over an area of 1652 ha. The average height of the hills varies between 400 to 1000 m and highest point in the valley i.e. Sakesar lies at 1527 m above sea level. The HM forest receives 600 mm of annual precipitation (mostly falling as monsoon) and mean annual temperature remains 24 °C. The dominant vegetation in the forest comprise of co-occurring bi-climax community of *A. modesta* and *O. ferruginea* with dense understorey shrub cover^18^. The forest is known for its high productive and protective values and is surrounded by vast tracts of agricultural fields and four towns. This reserved forest is under state control and is managed by the Forest Department of District Khushab, Pakistan (Fig. 1).

### NDVI classification

NDVI (Normalized difference vegetation index) time series maps (year 2007, 2013 and 2019) were developed by measuring the reflectance of red and infrared bands of satellite imagery based on density and intensity of vegetation using Eq. (1)^23^. The imagery was downloaded from United States Geographical Survey (USGS—Earth Explorer) website and was processed in ArcMap (v.10.2). The reference years were selected to analyze the state of forest in the pre and post implementation phase of the Forest Act (Punjab Forest [Amendment] Act 2010 in case of this study). To compare NDVI values, all satellite images were acquired for the same month of the respective years (i.e. October) after monsoon rains when vegetation was stable and also to avoid seasonal variations (Table 1). The difference in the dates of image acquisition (although in the same month) was unavoidable because of cloud cover in the satellite imagery and image distortion. It was also based on availability of satellite data for a particular date in the month of October.

1 $$NDVI \, = \, \left( {NIR \, {-} \, RED} \right)/\left( {NIR \, + \, RED} \right),$$

where NIR and RED are the reflectance in near infrared and red bands respectively based on the density of vegetation. The NDVI values generally range between − 1.0 and + 1.0 referring to no or high (healthy) vegetation cover. A value of 0.1 or below indicates bare soil or rock, however, value of 0.2 to 0.3 represents shrub/ low vegetation cover and higher values represent trees/high vegetation cover^24^.

**Table 1:** Detail of the satellite imagery processed for NDVI and LULC.

Sr. no.	Satellite/sensor	Date of imagery (yyyy/mm/dd)	Path/row	Resolution (m)	NDVI bands (Red and NIR)
01	Landsat 7 ETM +	2007/10/08	150/37	30	Band 3 and Band 4
02	Landsat 8 (OLI-TRIS)	2013/10/16	150/37	30	Band 4 and Band 5
03	Landsat 8 (OLI-TRIS)	2019/10/17	150/37	30	Band 4 and Band 5

### Valuation of ecosystem carbon storage and sequestration services

Integrated Valuation of Ecosystem Services and Tradeoffs (InVEST) carbon model (v. 3.3.3) was used for the quantification, mapping and valuation of the effect of LULC changes on ecosystem carbon storage and sequestration. The model was fed with LULC spatial data for year 2019 and carbon data from field estimates of four pools i.e. aboveground, belowground, soil and litter (dead) to estimate carbon stored in each grid cell. Carbon stock of the entire ecosystem was estimated by aggregating individual carbon estimates of each LULC class (Eqs. (2), (3)). Future LULC raster (for year 2030) was input into the model to predict carbon sequestration (Eq. (4)) in future^25^. For economic valuation, the country specific median price of a $36.67/ton of carbon (the social cost of carbon) with a discount rate of 2% was used^26^. The model resulted in three dimensional estimate of carbon stored and sequestered in each LULC class along with its monetary benefit.

2 $$C_{m,i,j} = \, A \, \times \, \left( {Ca_{m,i,j} + \, Cb_{m,i,j} + \, Cs_{m,i,j} + \, Cd_{m,i,j} } \right),$$

3 $$C=\sum \limits_{m=1}^{n}C m,i,j,$$

4 $$S \, = \, C^{fut} {-} \, C^{cur} ,$$

where A is the area of grid cell (0.09 ha), Ca_*m,i,j*_, Cb_*m,i,j*_, Cs_*m,i,j*_ and Cd_*m,i,j*_ refers to the aboveground, belowground, soil and dead (litter) carbon density (Mg ha^−1^) for grid cell (*i, j*) for land use type (*m*). C is the aggregate carbon density of the ecosystem for the current year (C^cur^) and predicted year (C^fut^) and S is the carbon sequestration across the study area^25^.

### InVEST input data preparation

#### Land use land cover map

Time series LULC maps of the study area were developed to spatially represent forest carbon stock through InVEST (v. 3.3.3) carbon model. After stacking bands of satellite images, land use type was categorized into five classes i.e. agriculture, built up area, barren land (with no vegetation cover) and *O. ferruginea* and *A. modesta* vegetation based on land uses inside and on the forest boundary. The built up area was represented by the shrine of saint Hayat-ul-Mir and few temporary huts around coal mining sites (Table 2). For each LULC class, a representative number of polygons were selected as training sample areas and supervised maximum likelihood classification was done to develop LULC maps of year 2007, 2013 and 2019. As *A. modesta* and *O. ferruginea* classes were most dominant (based on field data), therefore approximately 30–50 polygons were taken as training samples (randomly) for these two classes. The other three classes i.e. agriculture, built-up and barren land had comparatively much lower cover areas (based on field observations) therefore 10 polygons were taken for each class as training samples. Classification accuracies of these maps (based on ground truth data) were estimated to be 81%, 83% and 80% for year 2007, 2013 and 2019 respectively.

**Table 2:** Description of the LULC classes classified in this study.

LULC class	Description/definition
*O. ferruginea*	Area of the forest with cover of *O. ferruginea*
*A. modesta*	Area of the forest with cover of *A. modesta*
Agriculture	Areas with agriculture activity/cultivation
Built up area	Area with any settlements/houses/mining colonies/temporary huts/built up structures
Barren land	Area with bare rocks and no vegetation cover or settlements

The prediction of future LULC dynamics for year 2030 under business as usual scenario (BAU) was done through CA—Markov modeling. Markov chain analysis was implemented on time period 2013 to 2019 to create transition areas and transition probability matrix among the initial and final states to predict temporal LULC trends (Eqs. (5), (6)). Cellular Automata (CA) model was used for spatial simulations. In CA, each cell decided the data between different states in time for itself and its neighboring cells (through contiguity filter) and decided change in each cell by rules. The weight factor is applied based on proximity of nuclear and neighbor cells which is then combined with transition probabilities to predict the state of closer cells so that land use change is not a random decision. A combined CA–Markov model was used to simulate the spatio-temporal dynamics among the LULC classes using transition probability matrix as input to CA model to predict future LULC^11,25^.

$$P_{ij} = \left[ {\begin{array}{*{20}l} {p_{11} } \hfill & {p_{12} } \hfill & \cdots \hfill & {p_{1n} } \hfill \\ {p_{21} } \hfill & {p_{22} } \hfill & \cdots \hfill & {p_{2n} } \hfill \\ \cdots \hfill & \cdots \hfill & \cdots \hfill & \cdots \hfill \\ {p_{n1} } \hfill & {p_{n2} } \hfill & \cdots \hfill & {p_{nm} } \hfill \\ \end{array} } \right],$$

5 $$(0 \le Pij < 1\text{ and }\sum \limits_{j=1}^{n}Pij=1, i,j=\text{1,2},....n),$$

6 $$S \, (t + \, 1) \, = P_{ij} \times \, S(t),$$

where S is the status of land use at time t and t + 1; and Pij is the transition probability matrix.

**Figure 2:**
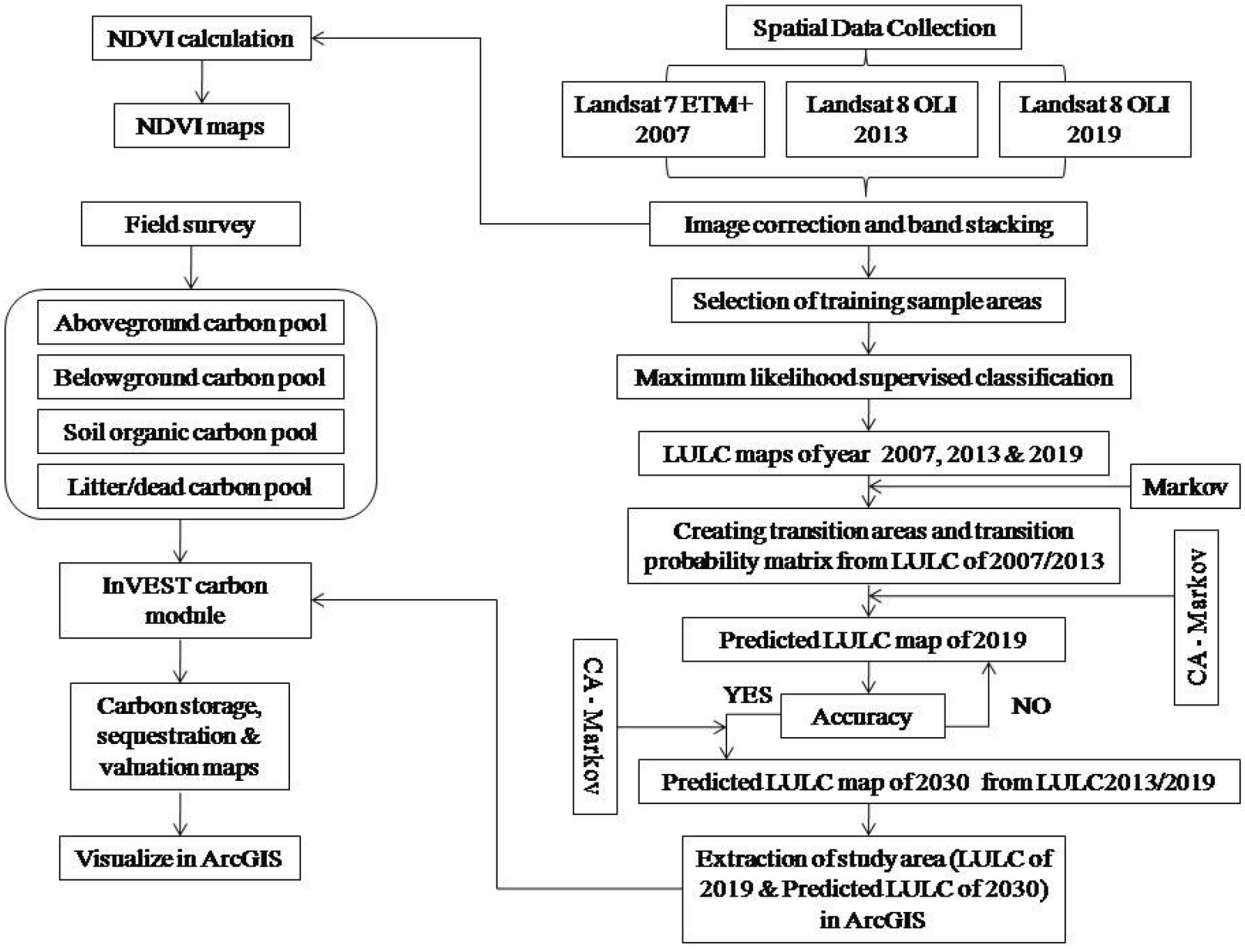
Methodological framework to determine NDVI and to produce LULC and ecosystem service maps.

Using CA–Markov model in the IDRISI Selva (v. 17.0), number of CA iterations was set to 11 as time interval, 5 × 5 contiguity filter and transition probability matrix (2013 to 2019) was used to predict LULC of 2030. Before running the final model, it was validated by creating a prediction map of 2019 from 2007 and 2013 LULC data and then it was compared with the actual (real) LULC map of 2019 using kappa statistics (Eq. (7)). The LULC maps generated were visualized in ArcGIS (v. 10.2), study area was extracted and converted to InVEST compatible file format before running sequestration and valuation model. The LULC change map was developed in ArcMap (v. 10.2) to visualize inter-conversion of LULC classes during 2019 to 2030. Complete scheme of study is presented in Fig. 2.

7 $$Kappa \, = \, \left( {Po \, {-} \, Pc} \right)/\left( {Pp \, {-} \, Pc} \right),$$

where kappa is index to test simulation accuracy; Po, Pc and Pp are the actual, expected and ideal (100%) simulation accuracies respectively^11^.

#### Assessment of forest carbon pools

Estimates of the above and belowground carbon stored in *A. modesta* and *O. ferruginea* were adopted from our recent study^19^ conducted in the HM forest by measuring tree dendrometric variables in 47 survey plots (0.04 ha). Sub-plots of 1 m^2^ were marked to collect leaf litter (dead fraction) and soil samples (at 15 cm depth). To determine carbon stored in the dead pool, 1 g of oven dried leaf litter was ignited at 550 °C in a muffle furnace for 2 h following Salehi et al.^27^. Soil total organic carbon (TOC) was determined through Walkley Black chromic acid wet oxidation method after reducing organic compounds of soil sub-sample (1 g) with 1 N K_2_Cr_2_O_7_. The unreduced dichromate was determined by oxidation–reduction titration. TOC (%) was multiplied with sample depth and soil bulk density to estimate soil organic carbon stock (SOC) on per hectare basis^28^.

### Statistical analysis

Statistical Package of Social Sciences (SPSS v.21) was used to compute the means and standard errors of the carbon stored in each LULC class. Multiple regression analysis was performed to predict carbon stored and area covered by each LULC class.

## Results

### NDVI cover

**Figure 3:**
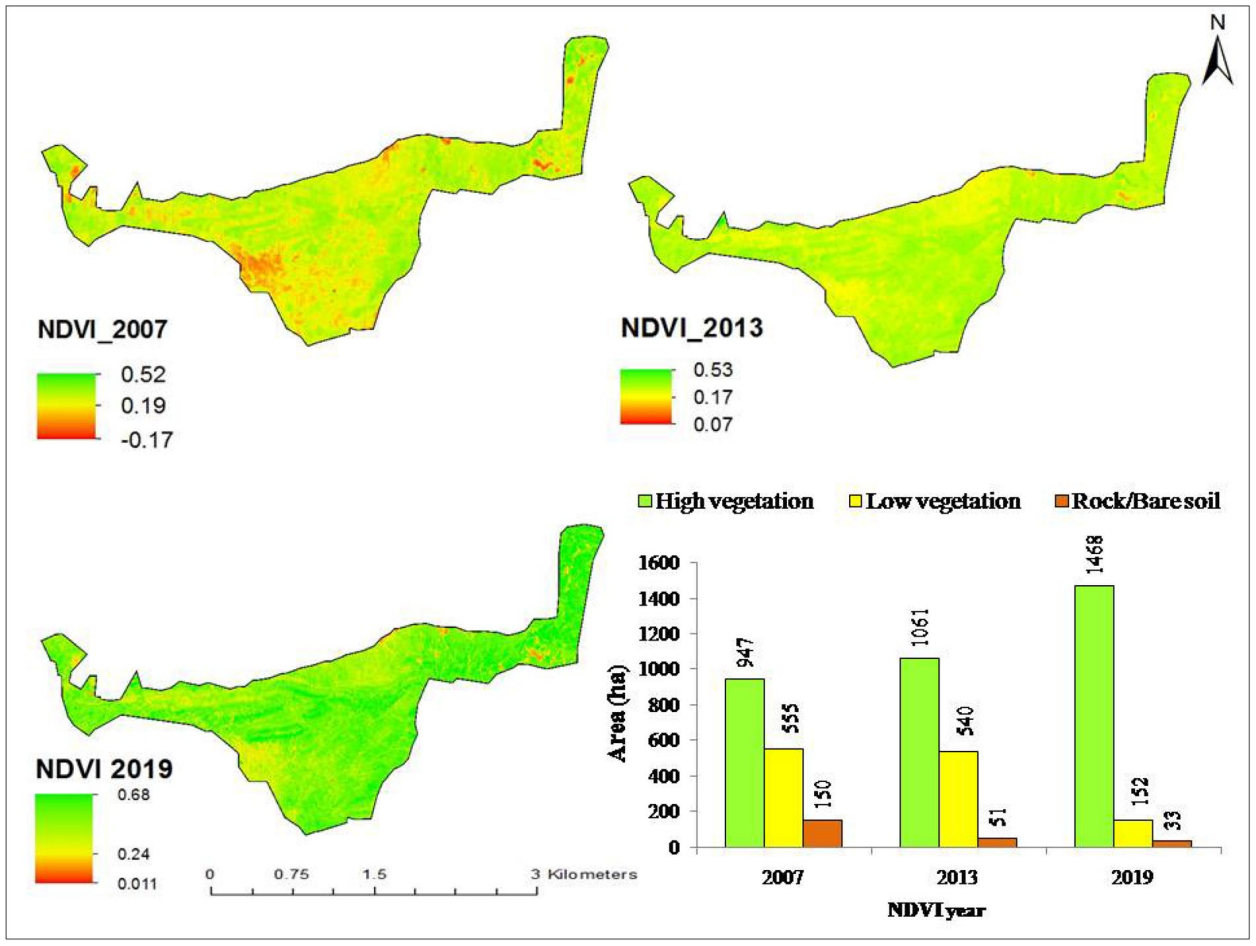
Time series analysis and area covered by each NDVI class in the HM forest (Software: ArcMap v. 10.2).

Figure 3 shows the time series Normalized Difference Vegetation Index (NDVI) of the HM forest. High vegetation (or tree cover) was found to be the most dominant NDVI cover class in the HM forest. The greenness index increased with time (or forest age) and highest NDVI (0.68) was calculated for trees covering an area of 1468 ha (89%) of the total 1652 ha in year 2019 compared to NDVI of 0.52 (947 ha or 57%) and 0.53 (1061 ha or 64%) for the same class in year 2007 and 2013 respectively. The irregularly distributed bare soil/rock represented change in NDVI value of − 0.17 to + 0.01 with decrease in its area from 150 ha (9%) in year 2007 to 33 ha (2%) in year 2019 respectively. In 2013 and 2019 most of the bare soil was replaced with vegetative cover either shrubs or climax species, except around mines, reducing the red reflectance. Change in low vegetation (shrub) cover was also observed during 2007 (555 ha or 34%) to 2019 (152 ha or 9%) and NDVI values varied from 0.19 to 0.24 representing the relative reflectance of red and infrared light.

### Land use land cover change

**Figure 4:**
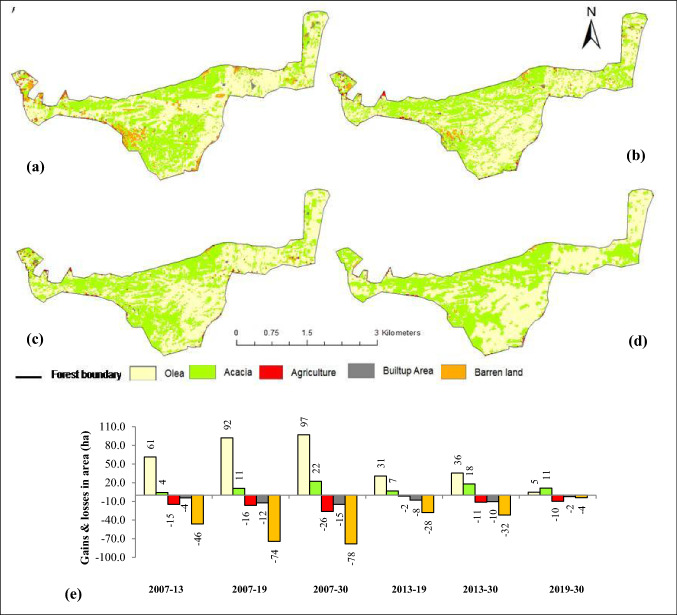
Land use land cover maps; (**a**) 2007, (**b**) 2013, (**c**) 2019 and (**d**) simulated BAU 2030 (**e**) gains and losses in area (ha) of LULC classes during 2007 to 2030 (Software: ArcMap v. 10.2 & IDRISI Selva v. 17.0).

Spatial and temporal trends in LULC changes during 2007, 2013, 2019 and simulated 2030 (under BAU scenario) are presented in Fig. 4 and Table 3. The Vegetation cover of the HM forest comprising of *A. modesta* and *O. ferruginea* gradually increased overtime during 2007 to 2030 decreasing the cover of other LULC classes i.e. agriculture, built up area and barren land. Most of the agriculture (32 ha) and built up area (19 ha) was found on the edges of the forest in 2007, while 81 ha of the land area was barren (no vegetation cover) with an irregular spatial distribution. Compared to the BAU 2030 LULC map, 78 ha, 28 ha, 32 ha and 4 ha in the barren land area was decreased between year 2007–2030, 2013–2019, 2013–2030 and 2019–2030, respectively. A similar trend in decrease in agriculture and built up area was observed for the same time frame. There was an overall gain in the area of *A. modesta* and *O. ferruginea* vegetation from 715 and 805 ha in year 2007 to 738 ha and 902 ha in the predicted year (2030) due to land cover conversions. The predicted LULC of 2030 represents very low area coverage of agriculture, built up and barren land on the edges of the forest only. The prediction accuracy of CA–Markov model was found to be 0.84 based on the kappa index of agreement between actual and simulated LULC of 2019.

**Table 3:** Area coverage of each LULC class in the HM forest during 2007 to 2030.

LULC classes	2007		2013		2019		2030	
	Area (ha)	Cover (%)	Area (ha)	Cover (%)	Area (ha)	Cover (%)	Area (ha)	Cover (%)
*O. ferruginea*	805	48.7	866	52.4	897	54.3	902	54.6
*A. modesta*	715	43.3	720	43.6	726	44.0	738	44.7
Agriculture	32	1.9	17	1.0	15	0.9	5	0.3
Built up Area	19	1.1	14	0.9	6	0.4	4	0.2
Barren land	81	4.9	35	2.1	7	0.4	3	0.2
Total area	1652	100	1652	100	1652	100	1652	100

### Carbon storage and sequestration service

In the HM forest, the highest amount of carbon was stored in the bi-climax community of *O. ferruginea* (15.2 ± 1.9 Mg/ha) and *A. modesta* (14.1 ± 1.8 Ma/ha) as aboveground carbon (AGC), belowground carbon (BGC), soil and dead carbon. The limited agriculture activity on the forest boundary was recorded with storing 3.8 ± 0.9 Mg/ha of carbon whereas least carbon was stored as SOC in the built-up and barren LULC classes (Table 4).

**Table 4:** Carbon stored (Mg/ha) in the LULC classes of the HM forest in year 2019.

LULC class	AGC	BGC	SOC	Dead C	Total C
*O. ferruginea*	8.7 ± 1.4	1.7 ± 0.02	4.8 ± 0.1	0.023 ± 0.001	15.2 ± 1.9
*A. modesta*	6.8 ± 1.1	1.4 ± 0.01	5.8 ± 0.4	0.012 ± 0.001	14.1 ± 1.8
Agriculture	1 ± 0.01	0.2 ± 0.001	2.6 ± 0.1	0.003 ± 0.0	3.8 ± 0.9
Built up area	–	–	1.1 ± 0.09	–	1.1 ± 0.09
Barren land	–	–	0.3 ± 0.001	–	0.3 ± 0.001

Multiple linear regression analysis revealed a significant positive relationship of LULC with total carbon stored (*p* < 0.05) in the pools of each class i.e. AGC, GBC, SOC and dead C. The analysis suggests increase in carbon storage with increase in the vegetation density of LULC class. Similarly, positive significant relationship was observed between LULC class and corresponding area covered (*p* < 0.001) (Table 5).

**Table 5:** Multiple linear regression analysis of LULC against area and carbon stored.

Variables	Regression equation	R^2^ adj.	F	Sig.
Total carbon	y = − 4.28x + 19.74	0.844	22.563	0.018
Area	Y = − 250x + 1080	0.761	61.386	0.000

**Figure 5:**
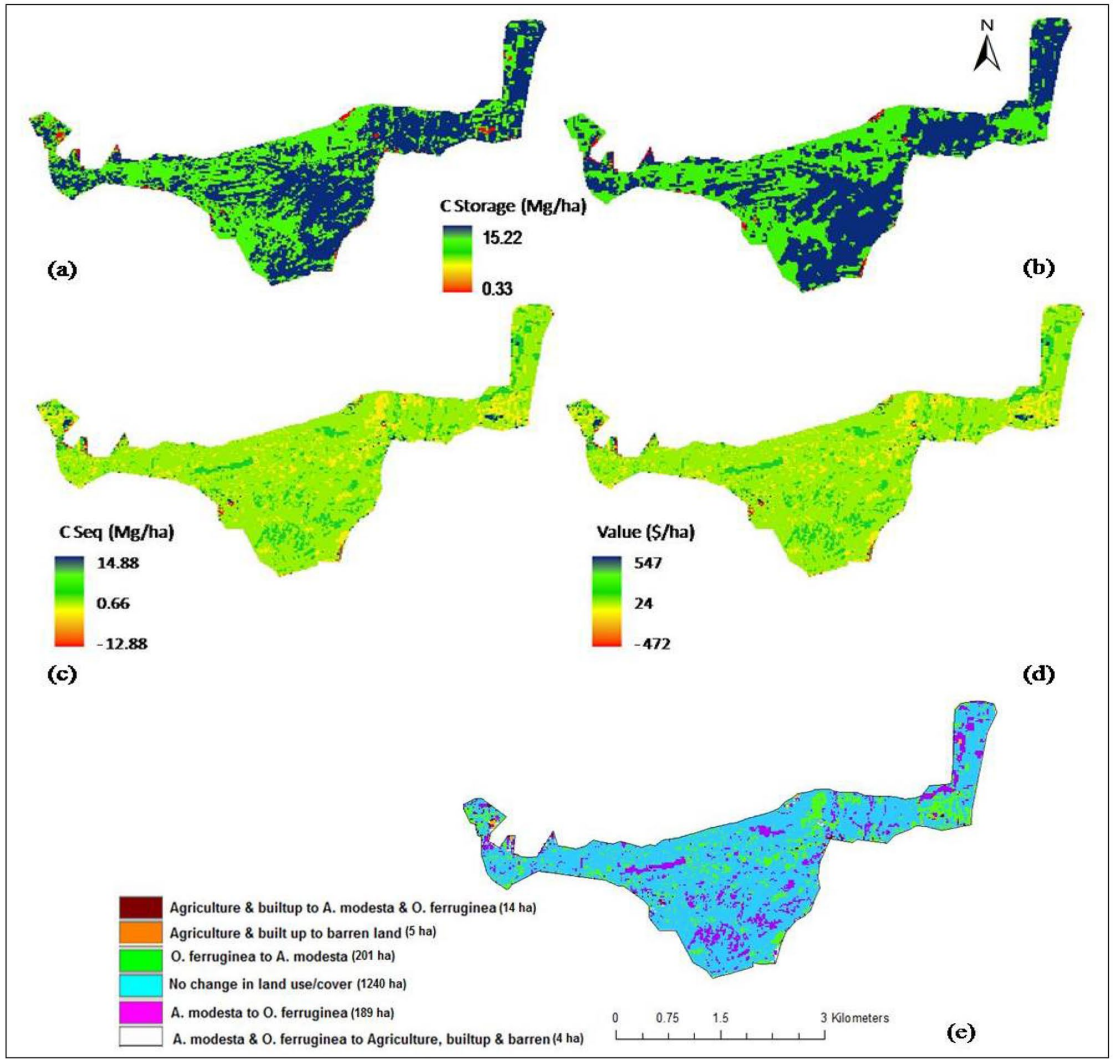
Spatial distribution of ecosystem carbon (**a**) storage in 2019, (**b**) in 2030, (**c**) sequestration, (**d**) its economic value in the HM forest and (**e**) LULC changes during 2019 to 2030 (Software: ArcMap v.10.2 & INVEST v.3.3.3).

The InVEST carbon model helped in identifying the LULC class with higher carbon storage potential. The tool estimated maximum 15.22 Mg/ha of total carbon stored in the vegetative cover of the forest in the base (2019) and predicted (2030) land covers. However, least carbon was stored (0.33 Mg/ha) in agriculture, built-up and barren land cover classes suggesting their least contribution in the provision of ecosystem service (Fig. 5a,b). Changes or interconversions in LULC classes between base (2019) and predicted (2030) year were detected to determine their benefit and potential for carbon sequestration (Fig. 5c–e). It was observed that under BAU scenario, ~ 14 ha of agriculture and built up area will convert into forest vegetation i.e. *A. modesta* and *O. ferruginea* in 2030. The InVEST carbon model predicted highest potential to sequester carbon (14.88 Mg/ha) with net 547 $/ha of monetary benefits for that area. The change analysis revealed that ~ 5 ha of agriculture and built up area will become barren in 2030. Additionally, 4 ha of vegetative cover will convert to other three LULC classes (agriculture, built-up and barren) emitting CO_2_ instead of carbon sequestration (− 12.88 Mg/ha) reducing overall net benefits (− 472 $/ha). No change in LULC for 1240 ha of land area was observed for which model did not calculate SR and benefits. However, interconversions of *A. modesta* and *O. ferruginea* classes were also observed. Cumulatively, the model calculated 23,912.18 Mg and 24,024.09 Mg of carbon stored in the HM forest currently (2019) and in future (2030) respectively, and 111.91 Mg of carbon sequestered with net present value (benefit) of $4112.05 at a discount rate of 2%.

## Discussion

Current study presents modeling based prediction of the expected LULC changes on regulatory ecosystem service of the HM forest on a spatio-temporal scale. The study site is under jurisdiction of provincial government and thus is a declared reserve forest under Punjab Forest (Amendment) Act 2010 prohibiting any cultivation, land clearings, grazing, mining and timber harvesting unless granted under limited rights^29^. Therefore, limited human activity was observed in the HM reserve forest except some agricultural activity and built up area on forest edges only. Barren land or area with no vegetative cover was seen throughout the forest, but it declined and was replaced with tree cover overtime (2007 to 2019) as also represented through NDVI.

NDVI helped in investigating density and health of the forest on spatio-temporal basis. The most dominant cover class in the HM forest was made by the bi-climax community of *O. ferruginea* and *A. modesta* represented with an increase in NDVI value overtime. The limited human activity in HM forest reserve resulted in increase (~ 32%) in its vegetation density and hence biomass carbon stock during 2007 to 2019. Reason for this increase in the vegetation density can be attributed to the implementation of Punjab Forest (Amendment) Act 2010. The act defined rights and restricted human interference in the form of encroachments, construction, timber and fuelwood harvesting, damage to soil and natural vegetation, cattle grazing, trespassing and any kind of land use change other than for the conservation purpose under sub-section 1 of section 26. The act also revised penalties to offences in the form of imprisonment for six months or fine ranging between PKR 10,000 to PKR 0.5 million or both depending on the worth of damage which could double if offence committed after sunset, as defined under sub-section 2–4 of section 26^29^. Consistent to the findings of present study, Kamwi et al.^30^ also reported increase in the forest cover of protected and communal areas of Zambezi region in Namibia during 1991–2010 compared to 1984–1991 after the implementation of forest policy restricting any human interference. In the absence of forest policy and participatory forest management, 37.38% decline in the forest area was observed in 2019 compared to 1991 (20%) due to agricultural expansions (+ 33.5%) in Komto protected forest of Ethiopia which is contrary to the findings of current study^31^.

Other reason for increase in the vegetated cover of HM forest is related to a significant reduction in the livestock grazing during the last 10 years due to shift to non-livestock dependent livelihood practices of the adjacent communities. This is evident from a significant increase in the number of factories from just 28 in year 2007 to 145 in year 2019 in the district. There was also a significant increase in the available agriculture machinery which reduced dependence on work animals^32–34^. The decrease in grazing pressure provided relief to the scrub forest vegetation (both shrubs and climax species) and it restored as reflected in the NDVI and LULC for year 2013 and 2019. Findings of this study are contrary to Mannan et al.^21^ reporting decrease in vegetation cover with lower NDVI values (1998 to 2018) for subtropical and moist temperate forests dominated with *A. modesta, O. ferruginea, Ziziphus* spp. and *Pinus* spp. on the foothills of the Himalayas, Pakistan due to increase in urbanization and human interferences. Zhao et al.^11^ however, reported increase in forest cover and carbon stock of Heihe River Basin in China during 2001–2015 after the implementation of the ecological engineering program.

With continued forest management practices and decreased grazing pressure, the cover of *A. modesta* and *O. ferruginea* is expected to further increase under BAU scenario (2030) replacing agriculture, built up and barren land covers. The shrine of Hayat-ul-Mir (representing built up area) will remain in place as it provides spiritual and cultural ecosystem services to the nearby communities of Kalial, Biyak and Khurra villages.

Apart from the LULC changes, other factors such as rising atmospheric CO_2_ concentration at a rate of 2.5 ppm/year (expected 438 ppm in 2030) could induce a fertilization effect enhancing the photosynthetic rate and supporting the growth of both shrub and climax species^35^. But, coupled with the elevated CO_2_ levels, future warming and change in precipitation could limit the stem and root growth with increase in soil respiration^36^. Ghafoor et al.^37^ reported significant reduction in the regeneration rate and growth of *A. modesta* and *O. ferruginea* in HM subtropical forest under short term climate warming. Considering the current pace of global warming, where already 1 °C rise in temperature has been observed over the last 40 years in Pakistan, decrease in precipitation in the Soan valley and future warmer climate with elevated CO_2_ concentration might result in decrease in ecosystem productivity^38,39^. But the model was limited in simulating the effect of climatic variability on land cover of the forest.

Gradual increase in tree cover during 2007–2019 and simulation for 2030 implies accumulation of biomass and higher carbon sequestration potential of the HM forest in the future. The order of carbon stored in the HM forest was as; aboveground > soil > belowground > dead pools. The aboveground vegetation was identified as the largest pool storing carbon where major contribution was made by *O. ferruginea,* but comparatively the estimates were lower than other subtropical forests of Pakistan as reported by Nizami (2012) and Shaheen et al.^40^. The estimates of soil carbon density of the current study are higher than those reported by Nizami^17^ and represent its significance in storing carbon for long term if undisturbed.

The biophysical assessment of tree carbon stock showed that the subtropical broadleaved evergreen species (*O. ferruginea*) stored more carbon than subtropical broadleaved deciduous species (*A. modesta*) in the HM forest reserve. This interspecific difference suggests the relative importance and potential of both species in carbon sequestration as indicated through the InVEST carbon model. A similar trend in relative carbon storage and sequestration was reported by Arunyawat and Shrestha^41^ for deciduous and evergreen plantations in Northern Thailand. The ecosystem service assessment presented an increase in carbon storage in the HM forest in the future under BAU scenario which is consistent to the findings of Sil et al.^8^ and Zhao et al.^11^ for the simulated period in the river basins in Portugal and China respectively. Carbon storage was found comparatively high at the sites of evergreen species than where deciduous species is distributed in the HM forest, whereas least carbon storage in the non-vegetated classes in the base and predicted year. The historical trend in the relative area coverage of both species during 2007–2019 and predicted LULC presented *O. ferruginea* dominating the *A. modesta* suggesting that under a future warmer climate the evergreen species will have more potential in mitigating climate change^37^. This analysis is also helpful in identifying areas of potential carbon storage and where to implement management interventions to increase the service of tree carbon sequestration.

The land use change analysis reported a large area of the forest (75%) where no change in LULC is expected during 2019 to 2030. The simplified carbon cycle in the InVEST carbon model assumed a linear change in carbon sequestration overtime and did not calculate SR where no change in LULC is expected to occur during base and predicted year. Therefore, the model resulted in a low sequestration estimate of 111.91 Mg C (or 0.006 Mg C/ha/year) during 2019 to 2030 for the remaining land area (25%) which is expected to undergo LULC change. Consistent to the present study, Zhao et al.^11^ also reported low sequestration rates due to model limitations.

The analysis also identified sites with negative sequestration rates where loss of stored carbon (as CO_2_ emissions) is expected to occur due to conversion of vegetated land to built up or barren area in future. Additionally, low SR was calculated for the land cover class where area under *O. ferruginea* is expected to be replaced by *A. modesta* on lower elevations. The low SR for this conversion is related to a relatively low potential of *A. modesta* (deciduous) to store carbon than *O. ferruginea* (evergreen) and InVEST assumed it as some loss of stored carbon. Increase in ecosystem service was observed where *A. modesta* is expected to be replaced by *O. ferruginea* on higher elevations in the future and it was highest for the pixels where agriculture, built up and barren area are expected to convert to forest vegetation.

Considering the intensity of LULC change in the HM forest, SR was low compared to the other studies. Sil et al.^8^ reported higher SR (1.63 Mg/ha/year) under projected (2020) forest expansion scenario in the Sabor river basin, Portugal due to 50% increase in broadleaved, chestnut and coniferous forests. The difference in the estimates of both studies is related to the type and extent of LULC converted and site climatic factors affecting plant growth. Compared to this study, Zhao et al.^11^ predicted 16% of the shrub land of the Heihe river basin in China to be converted into grassland and bare land in 2029 reducing the net carbon density. Leh et al.^12^ also documented decrease in ecosystem carbon storage and sequestration service during 2000–2009 from LULC changes in West Africa. Carbon density in a forest does not remain constant, but it fluctuates over the life cycle of a species with high sequestration ability during its full vigor growth phase and falls with tree age and thus also affects carbon additions to soil from litter production. Increase in site productivity by implementation of management interventions also improves ecosystem potential of carbon storage and sequestration^42^. Thus the oversimplification of the carbon cycle in InVEST model and assumption of zero SR where no LULC change is observed might lead to some misrepresentation of the actual supply of this ecosystem service.

Like carbon SR, gains and losses in the economic value of ecosystem service was observed for the type of LULC converted during 2019–2030. The monetary benefit of carbon sequestration is expected to be highest at the sites where non-vegetated cover classes might convert into *O. ferruginea* and *A. modesta* and decrease in it where the forest cover is expected to be replaced with barren land. The net present value (NPV) of carbon sequestered in the current and future land covers ($4112 or PKR. 0.66 million only) of the HM forest was low because of LULC change in 25% of the land area only and limitation of the model to calculate NPV on remaining 75% of the intact area. The monetary value of this ecosystem service is difficult to be compared with other studies due to the difference in currency units, the social cost of carbon (SCC) and discount rate reflecting society’s preference for immediate benefits of carbon sequestration over future benefits. Contrary to the findings of this study, Sil et al.^8^ with SCC of $ 23, $ 44 and $ 312 (at a discount rate of 1, 3 and 7%) reported a significantly high value of this ecosystem service from approximately 50% change in LULC under future scenario. Together with the estimates of carbon storage and sequestration, the economic valuation of this service can help in identifying the areas of potential carbon sequestration to maximize the net benefits of forest management interventions.

## Conclusion

The current study reveals that land cover changes have occurred in the Hayat-ul-Mir forest during past decade. Over time (2007 to 2019), the land covers of *A. modesta* and *O. ferruginea* were increased and are further expected to increase in the simulated period (year 2030) based on BAU scenario. The current forest management practices and the restrictions imposed by the act were found satisfactory in reducing human impact although limited agricultural activity on the forest edges was observed but in the declining trend. Built up and the barren land area covers also decreased overtime and most of these classes were represented around coal mining sites and shrine during current and simulated land covers. The biophysical and economic assessment of the service suggested increase in future carbon storage with current management practices. The InVEST model was limited in the assessment of carbon sequestration and did not calculate it for the area which is expected to remain same in 2030. This might had led to a misrepresentation of actual carbon sequestration potential and net benefits of the HM forest. Improvements in the model are therefore required to accurately account for the carbon sequestration rates. Some interconversions of the LULC classes were also observed for the simulated land cover where the vegetation might replace the other land uses. For those parcels of land, such interconversions would bring positive impact in terms of carbon storage. The model estimated negative rates for areas where termination of agriculture and mining activity (representing some built up area) would make land vacant. CO_2_ emissions are expected from those land parcels if not restored with native vegetation. Hence based on this analysis, the study provides a valuable input to inspect the land uses that are worse off and where appropriate management interventions must be introduced to increase the carbon storage and sequestration potential of the subtropical forest.

## Data Availability

The data generated and analyzed in this study is included in this article.
